# Public Health Interventions and SARS Spread, 2003

**DOI:** 10.3201/eid1011.040729

**Published:** 2004-11

**Authors:** David M. Bell, World Health Organization Working Group on Prevention of International and Community Transmission of SARS

**Affiliations:** * World Health Organization, Geneva, Switzerland

**Keywords:** SARS, interventions, quarantine, epidemiology, travel, travel medicine, policy review

## Abstract

The 2003 outbreak of severe acute respiratory syndrome (SARS) was contained largely through traditional public health interventions, such as finding and isolating case-patients, quarantining close contacts, and enhanced infection control. The independent effectiveness of measures to "increase social distance" and wearing masks in public places requires further evaluation. Limited data exist on the effectiveness of providing health information to travelers. Entry screening of travelers through health declarations or thermal scanning at international borders had little documented effect on detecting SARS cases; exit screening appeared slightly more effective. The value of border screening in deterring travel by ill persons and in building public confidence remains unquantified. Interventions to control global epidemics should be based on expert advice from the World Health Organization and national authorities. In the case of SARS, interventions at a country's borders should not detract from efforts to identify and isolate infected persons within the country, monitor or quarantine their contacts, and strengthen infection control in healthcare settings.

## Abstract 2

Screening at international borders should not detract from public health interventions to control SARS within a country.

The 2003 outbreak of severe acute respiratory syndrome (SARS) is a modern example of containing a global epidemic through traditional or nonmedical public health interventions. The interventions included finding and isolating case-patients; quarantining contacts; measures to "increase social distance," such as canceling mass gatherings and closing schools; recommending that the public augment personal hygiene and wear masks; and limiting the spread of infection by domestic and international travelers, by issuing travel advisories and screening travelers at borders. Some measures were implemented pursuant to recommendations of the World Health Organization (WHO); others were implemented by governments on their own initiative. A novel technology, infrared scanning, was used extensively in some countries to try to identify persons with fever at international borders and in public places. After the outbreaks, WHO sought information to help assess the effectiveness of interventions in preventing the transmission of SARS in the community and internationally. Of particular interest was information on the effectiveness of thermal scanning of travelers.

## Methods

Information was obtained by reviewing scientific literature and surveying members of an informal WHO working group about preventing community and international transmission of SARS. Members were surveyed with standardized questionnaires regarding measures taken in their countries and evaluation studies known to them. Preventing transmission in healthcare settings was not addressed but had a major impact on preventing the transmission of SARS into the community and internationally (*1**,**2*).

## Results

### Local and National Interventions

#### Identifying Patients and Quarantining Contacts

Ascertaining and isolating case-patients, combined with rapid identification and management of contacts, were highly effective in interrupting transmission in several countries (*1**–**6*).^2^ For example, a study in Singapore demonstrated a correlation between rapidly isolating patients after onset of symptoms and a decreased number of secondary cases among their contacts (*4*) (Figure). Contacts in these countries were placed in various forms of quarantine or, less commonly, monitored for symptoms without confinement and isolated if and when symptoms emerged. The location of quarantine was usually at home but was sometimes at a designated residential facility (e.g., for travelers, persons who did not wish to remain at home for fear of exposing their families, homeless persons, and noncompliant persons). In some cases, quarantined persons were allowed to leave the quarantine site with the permission of local health authorities if they wore masks and did not use public transportation or visit crowded public places. In at least one area, these restrictions were applied to essential workers and termed "work quarantine."

**Figure:**
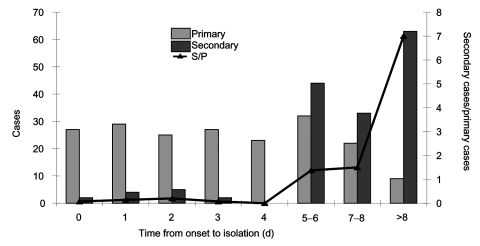
Severe acute respiratory syndrome cases in Singapore, February 25–May 5, 2003. Number of primary cases (light gray) by time from symptom onset to isolation, number of secondary cases infected by such cases (dark gray), and mean number of secondary cases per primary case. Reprinted with permission from Lipsitch M, Cohen T, Cooper B, Robins JM, Ma S, James L, et al. Science 2003;300:1966–70. Copyright 2003 by the American Association for the Advancement of Science. http://www.sciencemag.org

Several respondents emphasized that the modern concept of quarantine differs greatly from quarantine in past centuries. Quarantine is most acceptable and arguably most effective when protecting the health and rights of quarantined persons is emphasized. In previous centuries, sick and exposed persons were often locked up together and received limited medical care. Moreover, quarantine was sometimes applied in an arbitrary and discriminatory fashion, targeting lower socioeconomic classes and racial minorities. The modern concept emphasizes science-based interventions with attention to the medical, material, and mental health needs of quarantined persons and protecting fundamental human rights. Exposed persons who are not sick should be separated from symptomatic patients, monitored for the minimum time necessary (e.g., one maximum incubation period), and provided appropriate medical care at the first sign of illness during the monitoring period. Quarantine may be applied to individual persons, to small groups, or, in extreme cases, to entire neighborhoods or other geographic districts ("cordon sanitaire") (*7**,**8*).

In the SARS epidemic, persons under quarantine were mostly confined at home and actively monitored for symptoms. In several countries, quarantine was legally mandated and monitored by neighborhood support groups, police and other workers, or video cameras in homes. In other areas, compliance was "requested," but court orders were issued for a small percentage of noncompliant persons. Reports indicate that SARS was diagnosed in 0.22% of quarantined contacts in China-Taiwan, 2.7% in China-Hong Kong Special Administrative Region (SAR), and 3.8%–6.3% in China-Beijing. These different rates were partly due to different criteria for placing persons in quarantine. Contacts at highest risk (aside from healthcare workers with certain unprotected patient care exposures) had been exposed to ill family members (*6**,**9**–**11*).

Quarantine led to financial and psychosocial stresses, risk communication, compensation, and workforce staffing issues for persons, families, employers, and governments. Legal appeals and defiance of quarantine orders were rare (*2**,**6**,**8**–**13*).

The optimal management of contacts, stratified according to risk of becoming ill, remains under discussion in several countries, e.g., whether confinement is always needed or close monitoring of health status without confinement would suffice. Reports from Canada indicate that the insidious onset of symptoms sometimes posed challenges for clinicians and public health officials. "Timely diagnosis and isolation of cases were sometimes hindered by delays in patient recognition of symptoms, obtaining medical evaluation, and/or physician recognition of the significance of symptoms, which occasionally waxed and waned early in illness" (A. McGeer and D. Low, Mount Sinai Hospital Toronto, pers. comm.). "In Toronto, some healthcare workers continued to work without recognizing that they were ill, perhaps confusing their symptoms with fatigue, despite daily screening and repeated messages not to come to work if ill. This resulted in transmission to patients and staff" (B. Henry, Toronto Public Health, pers. comm.).

### Measures To Decrease Time from Symptom Onset to Isolation of Patients

Public campaigns to accelerate reporting and evaluating symptomatic patients appeared to decrease the interval between onset of symptoms and isolation of ill patients in several areas (3,4). Novel interventions included urging the entire population of affected areas to measure their temperature at least once daily, fever telephone hotlines (*14*), and fever evaluation clinics with appropriate infection control measures. Thermal scanning in public places was implemented in several areas where community transmission was suspected. Data on the effectiveness of this practice are not available, but in Beijing thermal screening was not an efficient way to detect cases among intercity travelers (*5*).

#### Measures To Increase Social Distance

Measures to increase social distance, e.g., canceling mass gatherings; closing schools, theaters, and public facilities; and requiring masks for all persons using public transport, working in restaurants, or entering hospitals, were implemented in areas where extensive unlinked community transmission of SARS coronavirus (SARS-CoV) was suspected. Many persons in these areas also chose to wear masks outside their homes. These measures were often applied simultaneously with other measures, including enhanced contact tracing, which makes their independent effectiveness difficult to assess. However the simultaneous introduction of a variety of measures was temporally associated with dramatic declines in new SARS cases. A case-control study in Beijing found that wearing a mask more frequently in public places may have been associated with increasing protection (*15*). Another case-control study in China-Hong Kong found that using a mask "frequently" in public places, washing one's hands >10 times per day, and "disinfecting living quarters thoroughly" appeared to be protective (*16*). The types of masks used were not specified. With the exception of the Amoy Gardens cluster in which SARS-CoV was apparently transmitted through accidentally produced aerosols of sewage (*17*), SARS transmission in the community from aerosols or in social settings appeared to be rare.

#### Disinfection

In some areas, disinfectants were applied inside the homes and vehicles of persons with SARS, ambulance tires, and pedestrian walking zones. Little information exists on the effectiveness of disinfectant use in reducing community or hospital transmission. In Hong Kong, disinfecting living quarters thoroughly (not otherwise defined and reported retrospectively by telephone) appeared to be protective (*16*).

### Measures for International Travel

#### Travel Advisories

Travel advisories (e.g., advice to postpone nonessential travel) were issued by WHO and various governments. Air travel to areas affected by the advisories decreased dramatically during the epidemic (M.A. Hinayon and D. Gamper, Airports Council International, communication to WHO), although the impact of advisories compared with other sources of information to travelers, such as news media reports of SARS cases, is difficult to assess.

#### Measures for International Borders

Passive and active methods were used to provide information and screen entering and exiting travelers. These methods included signs, videos, public address announcements, distributing health alert notices, administering questionnaires to assess symptoms and possible exposure, visual inspection to detect symptoms, and thermal scanning.

Few data exist on the relative effectiveness of methods of providing information to travelers. Available data on the effectiveness of screening and other measures directed to travelers are sometimes difficult to interpret because they may not distinguish between entry and exit screening, specify how many entering travelers were from affected countries, distinguish the epidemic period from subsequent, or include the number of SARS cases detected.

#### Health Alert Notices to Entering Travelers

Combined data from Canada, China (mainland, Hong Kong SAR, and Taiwan), France, Singapore, Switzerland, Thailand, and the United States indicate that approximately 31 million travelers entering these countries received health alert notices. Of these, approximately 1.8 million were reported as arriving from affected areas; this estimate is likely low given the difficulties in tracking travelers and the fact that many airline passengers change planes en route. Inadequate data exist to evaluate the effect of distribution of most of these notices. China-mainland reported distributing 450,000 notices and detecting four SARS cases that may have been linked to the notices (M. Song, China Dept of Health and Quarantine Supervision and Management, communication to WHO). Thailand reported having printed 1 million notices and detecting 113 cases of illness directly linked to the notices (108 at airports, 1 at a seaport, and 4 at land crossings). Twenty-four cases were suspected or probable SARS: all of which were detected at airports (S. Warintrawat, Ministry of Public Health, Thailand, communication to WHO).

#### Entry Screening

Preliminary data from a worldwide survey indicate that among 72 patients with imported probable or confirmed SARS cases, 30 (42%) had onset of symptoms before or on the same day as entry into the country and symptoms developed in 42 patients (58%) after entry (J. Jones, United Kingdom Health Protection Agency, communication to WHO). A small percentage of persons completing entry health declaration questionnaires in affected areas during the SARS epidemic were diagnosed with SARS (Table 1).

**Table 1:** Health declarations by entering travelers at international borders, March 1–July 15, 2003^a^

Area	No. completed declarations (millions)	No. reporting symptoms	No. reporting contact with SARS	No. with SARS detected by declarations
Canada	10	3,481	0	0
China-mainland	13.2	2,035	500	2 (both had SARS contact)
China-Hong Kong SAR^b^	19.3	2,380	NA	2 (both had symptoms)
China-Taiwan	1.0	5,287	NA	0
Singapore	1.9	Very low	0	0
Total	45.4	13,000	500	4

^a^SARS, severe acute respiratory syndrome; SAR, special administrative region. ^b^Includes border between China-Hong Kong SAR and China-mainland.

Results combined from Canada, China (including the mainland and Hong Kong SAR), and Singapore indicate that no cases of SARS were detected by thermal scanning among >35 million international travelers scanned at entry during the SARS epidemic (Table 2; data for Hong Kong SAR include travelers arriving from China-mainland). Temperature screening of 13,839,500 travelers entering or leaving Beijing by air, train, or automobile identified 5,097 patients with fever, of whom 12 had probable SARS. These 12 included 10 of 952,200 domestic airline passengers and 2 of 5,246,100 train passengers. None of 275,600 international travelers who underwent temperature screening had SARS (*5*).

**Table 2:** Thermal scanning of entering travelers at international borders, March 1–July 15, 2003^a^

Area	No. scanned (millions)	No. febrile by scan (confirmed orally)	No. SARS found by scanning
Canada	0.6	248 (215)	0
China-Mainland	13.0	4,070 (351)	0
China-Hong Kong SAR^b^	15.1	NA (451)	0
China-Taiwan	1.0	1,211 (0)	0
Singapore	6.0	5,200 (3,160)	0
Total	35.7	10,729 (4,177)	0

^a^SARS, severe acute respiratory syndrome; SAR, special administrative region. ^b^Includes border between China-Hong Kong SAR and China-mainland.

In China-Taiwan, incoming travelers from affected areas were quarantined; probable or suspected SARS was diagnosed in 21 (0.03%) of 80,813. None of these 21 was detected by thermal scanning when they entered Taiwan (*9*) (S.K. Lai, Taiwan Center for Disease Control, pers. comm.).

#### Exit Screening

After WHO recommended exit screening on March 27, 2003 (*18*), no additional cases from airline travel were documented from countries with screening. Combined data from China (Hong Kong SAR and Taiwan) indicate that among 1.8 million people who completed health questionnaires at exit, 1 probable case of SARS was detected. Combined data from Canada, China (Hong Kong SAR and Taiwan), and Singapore indicate that no cases of SARS were detected among >7 million people who underwent thermal scanning at exit (Table 3) (S. Courage, Health Canada, S.K. Lai, China-Taiwan Center for Disease Control; P.L. Ma, Hong Kong SAR China Dept of Health; and B.K.W. Koh, Singapore Ministry of Health, communications to WHO). In some areas, "stop lists" were used at borders to prevent persons on isolation or quarantine lists from exiting. Anecdotes suggest that exit screening may have helped dissuade ill persons from traveling by air but may have been more successful in dissuading local residents from traveling abroad than in dissuading ill travelers from attempting to return home.

**Table 3:** Exit screening of travelers at international borders, March 1–July 15, 2003^a^

Area	No. health declarations	No. thermally scanned	No. SARS
Canada	584,819	397,563	0
China-Hong Kong SAR^b^	700,000	2.5 million	0
China-Taiwan	1.1 million	1.0 million	1— by health declaration
Singapore	NA	4 million	0
Total	2.4 million	7.9 million	1— by health declaration

^a^SARS, severe acute respiratory syndrome; SAR, special administrative region. ^b^Includes border between China-Hong Kong SAR and China-mainland.

#### Transmission on Commercial Aircraft

Five commercial international flights were associated with transmission of SARS from patients with symptomatic probable cases to passengers and crew (*1*). Notification of exposed passengers and studies of transmission risk were greatly hampered by difficulties in identifying and tracing passenger contacts (*19**–**23*). In the most comprehensive investigation, involving three flights with extensive passenger tracing and laboratory confirmation of index and secondary cases, a wide range of risk was noted (Table 4). For flight 2, in which the secondary attack rate was 18.3%, the risk of infection was increased for persons seated close to the index patient, but most passengers who became infected were seated farther away, even though their individual risk was lower (*19*). In another study, one person with SARS, who had difficulty breathing but was not coughing, infected two other passengers. One of these sat in the row in front of the index patient but the other passenger sat four rows, plus a passageway, behind and on the opposite side of the plane (*20*). On nine flights arriving in Singapore, the incidence of transmission from passengers with SARS who had respiratory symptoms was estimated at 1 in 156 persons (*21*). A fourth study found no transmission to passengers seated near a patient who took multiple flights (*22*). In comparison, an influenzalike illness developed within 3 days in 72% of passengers in a plane containing a person with symptomatic influenza and grounded for 3 hours without ventilation (*24*). The risk for transmission of tuberculosis during a long flight was also increased among, but not limited to, passengers seated close to a highly infectious index patient (*25*).

**Table 4:** Rates of severe acute respiratory syndrome transmission on commercial aircraft^a^

Flight	Duration	Index patient(s)	No. infected/no. on plane (%)
1	90 min	1 presymptomatic	0/315 (0.0)
2	3 h	1 fever, cough	22/120 (18.3)
3	90 min	2 fever; 2 fever, cough	1/246 (0.4)

^a^Source: ref *19*.

## Discussion

SARS-CoV was contained in human populations in 2003 largely by aggressive use of traditional public health interventions (case finding and isolation, quarantine of close contacts, and enhanced infection control measures in settings where care was provided to persons with SARS, especially in healthcare facilities and homes). These measures also contained a smaller SARS outbreak in 2004 that originated from a laboratory-acquired infection (*26*). Measures to decrease the interval between onset of symptoms and isolation were effective in containing community transmission. The independent effectiveness of general community measures to increase social distance (in addition to contact tracing and quarantine) and improve hygiene and wearing masks in public places requires further evaluation.

Limited information exists on the relative effectiveness of methods of providing information on SARS (or other illnesses) to travelers. For inbound travelers who may have been exposed to SARS, such information should include what to do if symptoms develop and the need to inform healthcare workers who provide care for them in advance to take appropriate precautions. Entry screening of travelers by using health declarations or thermal scanning at international borders had little documented impact in detecting SARS cases. Exit screening appeared only slightly more effective; however, the possible value of these interventions in deterring travel by ill persons and building public and business confidence was not assessed. Preventing passengers with SARS from boarding aircraft would likely have reduced transmission of infection, but the most cost-effective ways to accomplish this are uncertain. The difficulties in identifying and tracing passengers exposed on aircraft highlight the need for public health authorities to have a mechanism for rapid access to passenger contact information. In the case of SARS, the data on border screening indicate that if resources are limited, interventions at a country's international borders should not detract from efforts to identify and isolate infected persons within the country, monitor and quarantine their close contacts appropriately, and strengthen infection control in healthcare settings.

In retrospect, although SARS-CoV was transmitted primarily through the respiratory droplet route, certain epidemiologic parameters facilitated its containment through public health interventions. Presymptomatic transmission was not observed. Infectivity in most patients was low at onset of illness and seemed to peak during week 2 of illness in association with maximal respiratory symptoms, when patients were often in the hospital. Virus transmission was primarily by respiratory droplets, with little natural airborne dissemination but some environmental spread. With some important exceptions (Hotel M and Amoy Gardens in Hong Kong), transmission occurred primarily in healthcare or household settings, with close person-to-person contact. Cases among children were uncommon, and children did not seem to be involved in transmission. Although the reproductive number for SARS (R_0_, the average number of new cases resulting from a single infection in a susceptible community) was approximately 2–4, contact tracing was facilitated by its relatively long serial interval (time between onset of symptoms in successive patients in a chain of transmission: mean 8–10 days) and incubation period (median 4–5 days). Most infections did not lead to further transmission, although a small number of "super-spreading" events occurred in which single unrecognized cases transmitted to many people, usually in hospitals or households, before appropriate infection control precautions were in place (*1*).

Traditional public health interventions will likely be required again to combat an emerging or reemerging infection for which specific antimicrobial drug therapy and vaccines are nonexistent or in short supply. For infections that are relatively less transmissible (e. g., SARS or a strain of avian influenza not fully adapted to human-to-human transmission), early and bold use of such interventions may contain transmission. For more readily transmissible infections (e.g., an emerging pandemic strain of influenza), they would not completely halt transmission but might "buy time" during a narrow window of opportunity during which an effective vaccine could be produced and other preparations made. For countries lacking specific countermeasures, such as drugs and vaccines, nonmedical public health interventions may be the only measures available to combat epidemics (*27*). Decisions regarding implementation should be based on expert scientific advice from WHO and national authorities; the epidemiologic features of the disease and available resources should be taken into account. This article does not address political and economic factors that may lead to calls for adopting certain measures or the economic and social consequences that may ensue, but governments will also consider such factors in their decisions.

The WHO SARS Scientific Research Advisory Committee has identified further research needs for SARS (*28*). Priorities include evaluating the effectiveness of public health interventions in terms of cases detected, cases prevented, costs, and alleviating public concerns; identifying ways to make quarantines and other restrictions more focused and less burdensome for persons and societies; assessment of how "leaky" restrictions can be before they become ineffective; and developing rapid diagnostic tests. Limitations of the information include that it was collected retrospectively, and in some studies, laboratory testing to confirm SARS-CoV infection was not performed. In the event of future outbreaks, these issues will need to be studied prospectively so that decisions can be based on the best scientific information.

## Footnotes

2 The term “isolation” is applied to ill people; “quarantine” is applied to persons who have been exposed but are not ill.
